# Efficacy of Natural Polymer Derivatives on Soil Physical Properties and Erosion on an Experimental Loess Hillslope

**DOI:** 10.3390/ijerph15010009

**Published:** 2017-12-22

**Authors:** Jun’e Liu, Zhanli Wang, Yuanyuan Li

**Affiliations:** 1 School of Geography and Tourism, Shaanxi Normal University, Xi’an 710019, China; liujune5@snnu.edu.cn; 2 National Demonstration Center for Experimental Geography Education, Shaanxi Normal University, Xi’an 710019, China; 3 State Key Laboratory of Soil Erosion and Dryland Farming on the Loess Plateau, Institute of Soil and Water Conservation, Northwest A&F University, Yangling 712100, China; li_yuanyaun@nwsuaf.edu.cn; 4 Institute of Soil and Water Conservation, Chinese Academy of Science and Ministry of Water Resources, Yangling 712100, China

**Keywords:** natural polymer derivatives, rainfall infiltration, soil aggregate, shear strength, soil loss

## Abstract

Raindrops disperse large soil aggregates into smaller particles, which can clog soil pores, cause soil crusting, reduce rainfall infiltration and increase soil loss. It was found that natural polymer derivatives were effective in improving soil physical properties and decreasing soil erosion on an experimental loess hillslope. This study investigated the effect of new natural polymer derivatives (Jag S and Jag C162) on soil properties, rainfall infiltration and sediment yield at four rates of sprayed polymers (0, 1, 3 and 5 g/m^2^), three rainfall intensities (1, 1.5 and 2 mm/min) and a slope gradient of 15° with a silt loam soil through simulated rain. The results showed that both Jag S and Jag C162 significantly increased the shear strength and improved the aggregates composition of the soil surface. The water-stable soil aggregates >0.25 mm increased from 9% to 50% with increasing rates of Jag S and Jag C162. Jag S and Jag C162 also effectively increased rainfall infiltration and final infiltration rate, and reduced erosion compared to controls without natural polymer derivatives added. However, higher rates of Jag S produced lower infiltration rates. Although both Jag S and Jag C162 effectively influenced soil physical properties and erosion, the effect of Jag C162 was more significant than that of Jag S.

## 1. Introduction

Drought and soil erosion are two environmental problems on loess hillslopes. Runoff, which forms when the rainfall intensity exceeds the infiltration capacity of the soil, is the direct cause of slope erosion by detaching and transporting displaced soil particles while flowing across the soil surface. Crust formation at the surface of cultivated soils exposed to the impact of raindrops is a common phenomenon, which is formed by the combined effect of physical disintegration of soil aggregates on the surface by the impact energy of raindrops and physicochemical dispersion of soil clays. It can disperse cultivated soils aggregates into micro-aggregates, reduce infiltration rate and increase runoff and erosion [1,2,3], which causes negative effects on the survival and growth of plants in arid and semiarid regions. Therefore, improving soil physical characteristics and reducing runoff by increasing rainfall infiltration can be considered a vital measurement for controlling water and soil loss. 

Some studies indicate that soil amendments such as gypsum and polymers can improve the stability of soil structure by electrostatic absorption of polymer molecules on the clay particles [4], thereby preventing crust formation, increasing infiltration and reducing surface runoff as well as soil erosion [5]. The application of various synthetic polymers and surfactants has been reported in the technical literatures. Botha et al. [6] investigated the influence of polymer PVA on the liquid–solid contact angles of a fine sandy soil. Pini and Vigna [7] found the formation of soil micro-aggregates by using two uncharged polymers, PVA and detrans, to study the interaction of water soluble stabilizing agents with soil particles. Floyd [8] studied PVAc emulsion as a soil conditioner. Three types of polyurethane were used in soil stabilization to improve the erosion resistance [9], and the result indicated that the polyurethanes improved both strength and erosion resistance significantly. 

Among these soil conditioners, polyacrylamide (PAM) was widely used and was a particularly effective polymer in improving soil aggregation, increasing water infiltration and preventing erosion [10]. About 400,000 ha of agricultural land has been treated each year with PAM in the USA [11]. Many researchers have indicated that the application of anionic polymer could significantly improve soil physical properties, namely increasing water-stable aggregates, reducing tensile strength, bulk density, surface compaction and clay dispersion, and delaying runoff formation and decreasing erosion and runoff [12,13]. However, Yu et al. [14] found that spreading granular PAM alone was only beneficial for reducing erosion but not for maintaining high infiltration rate (IR), and only the addition of gypsum with granular PAM was effective for increasing infiltration rate through simulated rain. Other researches also showed lower rates of PAM application decreased runoff while the higher rates increased runoff [15]. The effect of PAM was influenced by the clay content of soil. Increasing the clay content of soil improved the effect of PAM treatments, and negative impact on some coarser texture soils may exist [13]. Vacher et al. [13] found that application of PAM on the soil surface on steep slopes did not reduce runoff significantly, but application of polyacrylamide with gypsum was effective for runoff reduction.

Previous studies indicate that soil amendments are a promising way to improve soil physical properties and reduce soil erosion. However, the effect of soil amendments depends on its physiochemical properties, the mode of application and its quantity which also varies from time and soils. Tang et al. [16] showed that the negative effects of soil amendments were observed on barren or salty soils, and the application rate, either low or high contents of soil amendments, hardly improved soil properties and reduced soil erosion. Other researchers also treated the soil with different integrated chemicals to obtain better results. For example, in order to find better polymers, Wu et al. [17] applied polypropylene acid (PPA), polythene alcoholic (PTA) and urea-formaldehyde poly-condensate (UR) to China’s loess areas through indoor laboratory experiments and outdoor artificial rainfall simulations. The results indicated that the amendment applications could decrease the erosive forces of raindrops, increase the water-stable soil aggregates contents, reduce surface crusting and improve rainfall infiltration. Additionally, in order to pursue better polymers, Liu et al. [18] studied the effect of new polymer NPD on sheet erosion of experimental loess slopes through simulated rainfall. The results indicated that NPD effectively delayed the onset, reduced volume and sediment content of the runoff by significantly increasing the shear strength and the content of large aggregates from soil surface. Therefore, it is necessary to develop new and effective macromolecular polymers to regulate rainfall infiltration in order to reduce surface runoff and soil loss. Jag S and Jag C162 are two new natural polymer derivatives in our research, as SOLVAY polymers that are extracted from bean embryos. These are green chemicals showing no irritation and no known adverse effects on aquatic species on which they were tested. 

The objectives of this research were to: (i) determine the effects of Jag S and Jag C162, two new natural polymer derivatives, at different rates on soil loss and rainfall infiltration; (ii) reveal the mechanisms responsible for their effects by analyzing the proportions of water-stable soil aggregates of different sizes and shear strength after Jag S and Jag C162 were spread under simulated rainfall on experimental loess hillslope.

## 2. Materials and Methods

### 2.1. Soil and Polymers

The soil samples for testing were collected from Ansai County of the Loess Plateau (a typical region with hills and gullies). Ansai (109°19’ E, 36°51’ N) located in northern Shaanxi Province has a mean annual temperature of 8.8 °C and an annual precipitation of 500 mm. The soil was a silt loam (USDA) collected from the farming layer with a depth about 25 cm. The contents of organic matter, clay, silt and sand were 0.5%, 8.7%, 54.7% and 36.6% respectively. The *d*_50_ was 0.037 mm. The soil was air dried, crushed, well mixed and then passed through a 10-mm sieve to remove weeds and stones. The polymeric compounds tested were natural polymer derivatives, neutral polysaccharide (Jag S) and cationic hydroxypropyl polysaccharide (Jag C162), as SOLVAY polymers that are extracted from bean embryos, and both are easy to dissolve in water. Jag S and Jag C162 are both polysaccharide; however, Jag S is neutral, and Jag C162 consists of cationic hydroxypropyl and polysaccharide, respectively. In addition, lab studies showed that they were green chemicals that have shown no irritating or adverse effects on aquatic species, and were harmless to humans and can be used for preparations of personal care formulations. 

### 2.2. Experiments

Experiments were conducted in the Simulated Rainfall Hall of the State Key Laboratory of Soil Erosion and Dryland Farming on the Loess Plateau at the Institute of Soil and Water Conservation (Chinese Academy of Science and Ministry of Water Resources in China). A rainfall simulator system with a side-sprinkler was used to apply simulated rainfall. This rainfall simulator can be set to rainfall intensity ranging from 0.5 to 3.5 mm/min, by adjusting water pressure and nozzle sizes. The fall height of raindrop from the top to soil slope surface is 16 m. The uniformity of simulated rainfall is higher than 80%. The kinetic energy of raindrop to strike soil slope surface at rainfall intensities from 1 to 2 mm/min is about from 365 to 847 J/h·m^2^, and the diameters of raindrops range from about 0.25 to 0.375 mm.

Experimental plots were constructed with metal frames of 1.2 m (length) × 0.4 m (width) × 0.25 m (depth), with adjustable slope gradients by a movable base. A metal outlet at the lower end allowed the collection of runoff samples. In the bottom of the plots, natural sand to a depth of 5 cm and overlaid with permeable gauze was used to drain the infiltration water. The soil was packed to a depth of 20 cm in four 5-cm layers at a bulk density of 1.2 g/cm^3^ (measured by a cutting ring in a compacted state). Before packing, the water content of the soil was adjusted to 14%, the typical level during the flood season on the Loess Plateau when most erosion occurs. After the soil was packed, Jag S and Jag C162 solutions of 1, 3 and 5 g/m^2^ were prepared in 2 L of water to produce final rates of 0.024%, 0.072% and 0.12%, respectively. These solutions were uniformly sprayed on the surfaces of the plots and the control plot was sprayed with equal water (2 L). The simulated rainfall experiments began approximately 15 h later. Four rates (0, 1, 3, and 5 g/m^2^), three rainfall intensities (1, 1.5, and 2 mm/min), and a slope gradient of 15°, which was setup based on the middle slope of cultivated land ranging mainly from 5° to 25° in the Loess Plateau, were tested with two replicates. The duration of all simulated rainfall events was 40 min.

### 2.3. Measurements

For each treatment, runoff samples were collected 1 and 3 min after the onset of runoff and then every 3 min until the end of the experiment. The runoff volumes were measured with a graduated cylinder [18], and the sediments were dried at 105 °C for 10 h and weighed to estimate the soil erosion rate by calculating the sediment weight per unit area per unit time and amount of rainfall infiltration by the principle of water balance, while the rainfall infiltration rate was defined as infiltration amount per unit time. The cumulative infiltration (mm) was defined as the sum of the infiltration rate (mm/min) multiplied by time in all the time during rainfall process, and the cumulative erosion modulus (kg/m^2^) was defined as the sum of the erosion rate (kg/m^2^·min) multiplied by time in all the time during rainfall process. Aggregate size distribution on the surface (0–1 cm) were measured by wet sieving [19] after rainfall in the classes of >5, 2–5, 1–2, 0.5–1, 0.25–0.5 and <0.25 mm. Each class of aggregates was dried and weighed. Three samples were measured for each treatment and averaged. After each simulated rainfall, six measurements of the shear strength of the soil surface were also taken using a 14.10 Pocket Vane Tester (Eijkelkamp, Giesbeek, The Netherlands). Because shear strength is closely related to water content, we measured the water content continuously after each simulated rainfall. Soil water content was measured by alcohol burning method [20], and three samples were taken from surface 1 cm soil. The final shear strength was measured when the water content dropped to 22–25% after air drying. All data were analyzed using SPSS (Chicago, IL, USA) by one-way ANOVA and least significant difference (LSD) tests. The significant level was 0.05.

## 3. Results

### 3.1. Effects of Jag S and Jag C162 on Soil Physical Properties

#### 3.1.1. Effects on >0.25 mm Water-Stable Aggregates

Coote et al. [21] suggested soil shear strength and aggregate stability as erosion indicators. Soil aggregates and shear strength of the topsoil are two important factors in soil erosion, so we used shear strength and the contents of water-stable aggregates >0.25 mm as the main evaluation criteria and indicators of soil structure. Soil characteristics play a dominant role in erosion as erodibility indexes. In addition to erosion forces (e.g., rainfall and wind), topography (e.g., slope, slope length, slope shape, and slope section), and surface conditions (roughness and covering), the ability of soil to resist the dispersion of rainwater and runoff is closely associated with soil erosion. 

**Figure 1:**
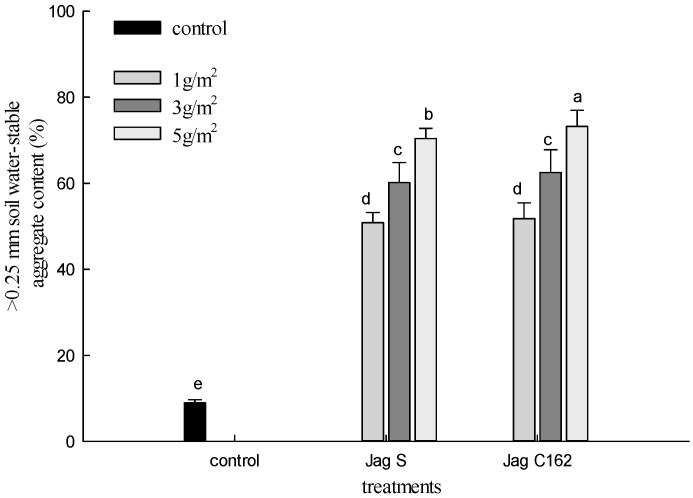
Effect of Jag S and Jag C162 on >0.25 mm soil water-stable aggregates. The letters “a”–“e” represent significant differences.

The effect of application rates of Jag S and Jag C162 (0, 1, 3 and 5 g/m^2^) on >0.25 mm soil water-stable aggregate content is presented in Figure 1. After applying Jag S and Jag C162 to the soil surface, the amended soil had a significantly higher content of >0.25 mm soil water-stable aggregates than the control sample. The percent mass of >0.25 mm soil water-stable aggregates of the untreated soil was only 9%, while the percentage increased to 50.85%, 60.19% and 70.41% in the three treatments of Jag S and 51.82%, 62.52% and 73.23% for Jag C162, respectively. Additionally, under the same application rates, the content of >0.25 mm soil water-stable aggregates after spraying Jag C162 was higher than that of spraying Jag S by 0.97%, 2.33%, 2.82%, respectively. This indicates that Jag S and Jag C162 both effectively promoted the aggregation of small particles into larger particles, and the positive effects of Jag C162 on the >0.25 mm soil water-stable aggregates are better.

In addition to the total content of >0.25 mm soil water-stable aggregates, the improvement of soil water-stable aggregates in different sizes was similar (Table 1), which increased by 0.26–24.9% significantly with the application of Jag S and Jag C162 compared with the control. The percentage mass of >5 mm, 2–5 mm, 1–2 mm soil water-stable aggregates with the application of Jag S and Jag C162 at three rates was higher than those of Jag S. The improvement of the soil water-stable aggregates in the 0.25–0.5 mm, 0.5–1 mm and 1–2 mm classes was significant, especially at rates of 3 and 5 g/m^2^, and the effect of Jag C162 treatments on the >1 mm soil water-stable aggregates was better than that of the Jag S treatments under the three rates. The results indicated that the new natural polymer derivatives Jag S and Jag C162 significantly promoted aggregate formations, and effectively facilitated the aggregation of small particles into larger particles because of the binding capacity of the polymers, which prevented the rainfall impacts and runoff from detaching the soil particles, in the same way as polyacrylamide [5].

#### 3.1.2. Effects on Shear Strength

**Figure 2:**
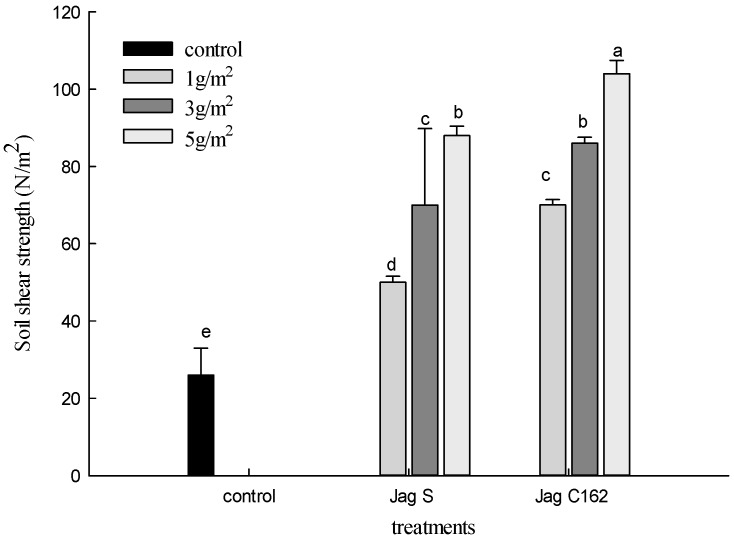
Effects of Jag S and Jag C162 on soil shear strength. The letters “a”--“e” represent significant~differences.

Soil shear strength refers to the ability or capability of a particular soil in a particular condition to resist or endure an applied force [22], which is another important factor for comprehensive abilities of controlling water erosion. The effects of Jag S and Jag C162 on soil shear strength are presented in Figure 2. 

Obviously, the application of Jag S and Jag C162 was effective in producing higher soil shear strength compared with the control at the three rates, and higher rates produced better improvement in soil shear strength. However, the effect of Jag S application was lower in improving the soil shear strength than that of Jag C162 application under the same conditions. Table 2 indicated that after 40 min of rain, the average soil shear strength at 1 g/m^2^ Jag S and Jag C162 increased by 92.9% and 173.3%; by 171.5% and 232.9% at 3 g/m^2^ and reached up to 241.7% and 305.4% at 5 g/m^2^, respectively. It is evident that the shear strength of soil treated with Jag C162 was 61.4–80.4% higher than that of Jag S treatments. This shows that Jag S and Jag C162 had an obvious effect of enhancing the stability of soil particles and resisting the damage from rainfall and runoff to reduce soil erosion effectively. 

### 3.2. Effects on Soil Erosion Process

#### 3.2.1. Effects on Rainfall Infiltration

**Figure 3:**
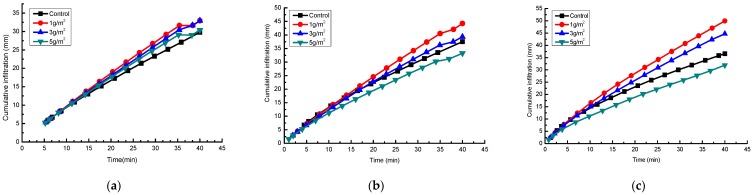
Cumulative infiltration of spraying Jag S under three rainfall intensities. (**a**) 1.0 mm/min; (**b**) 1.5 mm/min; (**c**) 2.0 mm/min.

**Figure 4:**
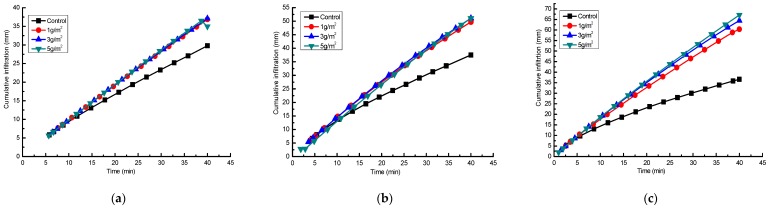
Cumulative infiltration of spraying Jag C162 under three rainfall intensities. (**a**) 1.0 mm/min; (**b**) 1.5 mm/min; (**c**) 2.0 mm/min.

Changes in cumulative infiltration on an experimental loess hillslope with rainfall time for Jag S and Jag C162 applications at 1, 3 and 5 g/m^2^ under rainfall intensities of 1.0, 1.5 and 2.0 mm/min are shown in Figure 3 and Figure 4, respectively. 

The cumulative infiltration increased linearly with the increase of rainfall time and intensity for all experiments, and compared with the control, the application of Jag S and Jag C162 improved the rainfall infiltration effectively, but the most effective rate of application varied with kinds and rates of the polymers. The infiltration rate decreased with increasing Jag S rate under the same rainfall intensity. After 40 min of rain, the cumulative infiltration remained relatively high when Jag S was applied, except that at the treatment rate of 5 g/m^2^ under 1.0 mm/min. Jag C162 also significantly improved rainfall infiltration, and higher rates of Jag C162 correlated with better cumulative infiltration under the same conditions. It is suggested that a moderate rate (1–3 g/m^2^) of Jag S treatment was more effective to prevent crust formation and promote aggregation on the surface to facilitate infiltration. This shows that it is very important to identify the effective threshold of the rates and choose the appropriate polymer to improve soil properties.

The final IR in Table 3 highlighted a quantitative comparison among the effects of the different treatments on soil susceptibility to crust formation. Applying either of the two polymers (Jag S and Jag C162) to the soil had a significant effect on the final IR at different rainfall intensities (1, 1.5 and 2 mm/min) on experimental loess hillslope. In the control treatment, the soil generated the lowest final IR (0.65, 0.73, and 0.59 mm/min) and the largest runoff amounts because of crust formation. However, spraying Jag S and Jag C162 on the soil surface increased the final IR by as much as one to two times, with values ranging from 0.70 to 1.51 mm/min compared with untreated samples. Under the Jag S treatment, the final IR decreased with increasing Jag S application rates, whereas the final IR increased as the amount of Jag C162 increased, because compared with Jag C162, Jag S is not as soluble, and a higher rate caused a more viscous solution resulted in blocking the soil porosity and reducing the final IR after spraying Jag S into the soil. Besides, spraying Jag C162 resulted, in most cases, in significantly larger final IR values (0.85–1.51 mm/min) than those obtained when spraying Jag S, which indicated improvement of final IR by applying Jag C162 was better than an application of Jag S.

#### 3.2.2. Effects on Sediment

**Figure 5:**
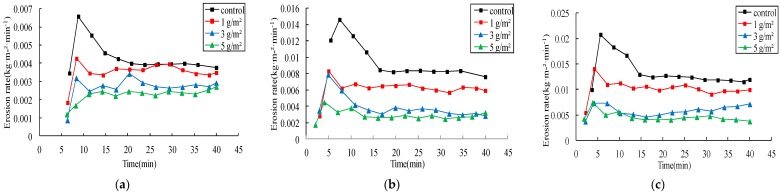
Erosion rates of spraying Jag S under three rainfall intensities. (**a**) 1.0 mm/min; (**b**) 1.5 mm/min; (**c**) 2.0 mm/min.

**Figure 6:**
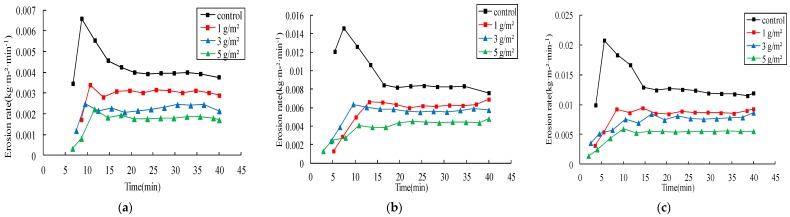
Erosion rates of spraying Jag C162 under three rainfall intensities. (**a**) 1.0 mm/min; (**b**) 1.5 mm/min; (**c**) 2.0 mm/min.

Jag S and Jag C162 increased the stability of soil particles and resisted the damage from rainwater and runoff, so they could effectively reduce sediment yield. The erosion rates and reductions of erosion modulus are presented in Figure 5 and Figure 6 and Table 4, respectively. Spraying Jag S and Jag C162 on the soil surface was effective in significantly reducing soil loss compared with the control. The erosion rates decreased with the increasing rates of both Jag S and Jag C162. The average reduction of cumulative erosion modulus at 1 g/m^2^ Jag S and Jag C162 was 23% and 34%, 49% and 43% at 3 g/m^2^ and reached up to 60% and 59% at 5 g/m^2^ under different rainfall intensities, respectively. Similar observations were reported in previous studies that also explored the effects of spraying granular PAM with gypsiferous material on loess soil [14,16]. However, the differences between the effects of Jag S and Jag C162 on erosion modulus were as follows: (1) the reductions of erosion modulus decreased as the amount of Jag S increased, while conversely, the higher Jag C162 application rate was more effective in increasing the reduction of erosion modulus; (2) the effect of Jag C162 was significantly higher than that of Jag S under the same conditions. Thus, the results indicated that both Jag S and Jag C162 were able to reduce erosion modulus, and the polymer Jag C162 was more effective.

## 4. Discussion

Many researchers have shown that polymers significantly improve soil physical properties. The viscosity of polymers can improve soil structure and increase the stability of aggregates [23]. Aggregates and their stability determine the sizes and stability of soil pores. Water-stable aggregates >0.25 mm can increase soil permeability and are an important indicator of anti-erosion [24], but also indicate soil quality. High aggregate stability can maintain more appropriate pore spaces for permeability and can resist erosion better. Aly and Letey [25] found that the effect of a polymer often depended on its capacity to facilitate flocculation, which prompted the polymer molecules to adhere on the surface of soil particles and act as a bonding agent, keeping soil particles together against the destructive impacts of water drops, and to diminish the destruction of the aggregates on the soil surface [26].

Our data showed that Jag S and Jag C162 could improve soil physical properties with their applications. The polymer molecule serves as a bridge between two soil particles in an aggregate by bonding with particles [27], so they can strengthen the interactions between soil particles and enhance the stability of aggregates. Thus, the aggregate size classes increased, more for large aggregates than for small aggregates, and the aggregation of small particles into larger particles was promoted, consistently with the findings by Xu et al. [28]. They reported that both synthetic and natural polymers had highly significant effects on aggregate size classes, especially for aggregates >2.0 mm, and could increase the mass of macro-aggregates and decrease the mass of micro-aggregates. The properties of polymers and their application rates both influenced their efficacy in soil [29]. Higher Jag S and Jag C162 rates increased more aggregates >0.25 mm and higher shear strength due to stronger adhesions. The soil shear strength can also serve as an indicator of soil resistance capacity during erosion [30].

Infiltration and erosion are closely associated with soil properties. Increases in aggregate sizes can lead to the maintenance of appropriate spaces for infiltration, thereby decreasing surface runoff. Changes in aggregate size distribution and shear strength can lead to the stabilization of the soil surface against shear-induced detachment [31], thereby decreasing erosion [32]. The applications of Jag S and Jag C162 changed the aggregate size distribution and shear strength which can effectively reduce soil crust formation, limiting surface runoff and runoff-induced erosion, similarly to polyacrylamide [33]. In this experiment, Jag S and Jag C162 were effective in improving soil shear strength and aggregates, thereby increasing rainfall infiltration and reducing soil loss on experimental loess hillslope. The effects were better as the rates increased. 

Applying the two polymers (Jag S and Jag C162) to the soil had a significant effect on the infiltration and the final IR at different rainfall intensities (1, 1.5 and 2 mm/min) on an experimental loess hillslope. In the control treatment, the soils generated the smallest final IR (0.65, 0.73, and 0.59 mm/min) and the largest runoff amounts because of crust formation. However, spraying Jag S and Jag C162 on the soil surface increased the final IR by as much as one to two times, with values ranging from 0.70 to 1.51 mm/min compared with untreated soils.

However, the results also showed that the effects of Jag S on the soil physical properties and soil erosion were lower than those of Jag C162. One possible explanation was that the effect of the polymer in stabilizing soil aggregates could be related to the capability of the polymer to move into aggregates. High rates of Jag S micro-molecules were hard to diffuse into the soil because of low viscosity, which limited the movement of Jag S molecules into soil aggregates, and too high rates of Jag S could jam the soil porosity so that the interaction between soil particles and Jag S was not sufficient, resulting in delaying the formation of more soil aggregates. In addition, a crust would form easily to decrease rainfall infiltration when the aggregates on the surface were broken by raindrops. Thus, the effect of Jag S on the soil physical properties and soil erosion was lower. 

Similar observations of improving rainfall infiltration and aggregates with the application of PAM were reported by Mamedov et al. [34], who found that the application of PAM resulted in increasing rainfall infiltration and aggregate stability compared with the control. Macromolecular polymers such as PAM can improve soil physical conditions. The polymers bind the soil particles and prevent dispersion due to their cohesiveness, thereby forming aggregates. These aggregates resist the effects of raindrops and soil crusting or sealing, which results in greater rainfall infiltration. Schamp et al. [35] explained that polymers could enhance the stability of aggregates via adhesion and adsorption. Shainberg and Levy [12] revealed that increasing aggregates’ stability could prevent soil crusting, and that polymer treatments could effectively decrease the formation of soil crusts by increasing aggregates’ size distribution and improving aggregates stability [27]. Compared with the control, the application of Jag S and Jag C162 significantly increased the mass proportions of different sizes of soil aggregates, especially for the >0.25 mm aggregates. In addition, Jag S and Jag C162 acted as binding agents that stabilized the soil aggregates, resisted soil crusting and increased infiltration; the result was consistent with previous studies (just like PAM) [5].

## 5. Conclusions

In conclusion, the above results indicated that polymers Jag S and Jag C 162 can effectively change the structure of the soil surface, maintain a high infiltration rate to limit runoff, reduce the detachment and transportation of soil particles and ultimately reduce erosion. Simultaneously, the resistance to erosion can be enhanced by increasing binding capacity between particles and by the higher mass percentage of water-stable aggregates. These results indicated that Jag S and Jag C 162 have broad potential applications for increasing rainfall infiltration and decreasing soil erosion by improving soil physical properties on loess slopes, and that the effect will be more effective when this technique is applied with traditional methods of soil and water conservation. However, we examined only a simple slope and three limited rates at three rainfall intensities, so the effects under more complicated conditions remain unknown. The improvement of rainfall infiltration thus requires more comprehensive studies and discussions. Further researches should be performed under different conditions, polymeric rates, soils and application modes. The effective thresholds and optimal doses of these macromolecular polymers should also be identified and quantified.

## Figures and Tables

**Table 1:** Increase of the content of different sizes of soil aggregates.

Sizes	Contents of Different Sizes of Soil Aggregates (%)							Percentage Increase of the Content of Different Sizes of Soil Aggregates (%)					
	Control	Jag S Rates			Jag C162 Rates			Jag S Rates			Jag C162 Rates		
(mm)		1 g/m^2^	3 g/m^2^	5 g/m^2^	1 g/m^2^	3 g/m^2^	5 g/m^2^	1 g/m^2^	3 g/m^2^	5 g/m^2^	1 g/m^2^	3 g/m^2^	5 g/m^2^
>5	0 e	0.26 de	0.52 de	1.74 c	0.88 d	3.43 b	5.92 a	0.26	0.52	1.74	0.88	3.43	5.92
2–5	1.06 d	3.72 cd	6.75 c	22.4 ab	10.8 c	20.2 b	25.9 a	2.66	5.69	21.3	9.70	19.1	24.9
1–2	1.86 d	12.0 c	16.1 ab	19.1 a	14.7 bc	16.3 ab	17.5 ab	10.1	14.2	17.2	12.9	14.4	15.7
0.5–1	2.47 c	14.1 ab	16.2 a	13.2 ab	11.4 b	10.4 b	10.0 b	11.6	13.7	10.7	8.96	7.93	7.55
0.25–0.5	3.58 c	21.4 a	20.1 a	14.0 b	14.3 b	11.6 b	13.8 b	17.8	17.0	10.4	10.7	7.98	10.2
<0.25	91.0 a	49.2 b	39.8 c	29.6 d	48.2 b	37.5 c	26.8 e	−41.9	−51.2	−61.4	−42.8	−53.5	−64.3

Notes: The letters “a”–“e” represent significant differences.

**Table 2:** Effect of Jag S and Jag C162 on soil shear strength.

	Control	Jag S Rates			Jag C162 Rates		
		1 g/m^2^	3 g/m^2^	5 g/m^2^	1 g/m^2^	3 g/m^2^	5 g/m^2^
Shear strength (N/m^2^)	26.0 e	50.0 d	70.0 c	88.0 b	70.0 c	86.0 b	104.0 a
Percentage increase (%)		92.9	171.5	241.7	173.3	232.9	305.4

Notes: The letters “a”–“e” represent significant differences.

**Table 3:** The final infiltration rate (IR) under different rainfall intensities.

The Final IR under Different Rainfall Intensities (mm/min)							
Rainfall Intensity (mm/min)	Control	Jag S Rates			Jag C162 Rates		
		1 g/m^2^	3 g/m^2^	5 g/m^2^	1 g/m^2^	3 g/m^2^	5 g/m^2^
1.0	0.65 c	0.81 ab	0.78 ab	0.72 bc	0.85 a	0.86 a	0.87 a
1.5	0.73 c	1.06 ab	0.86 bc	0.70 c	1.04 a	1.08 a	1.14 a
2.0	0.59 b	1.27 ab	0.85 ab	0.64 ab	1.03 ab	1.31 ab	1.51 a

Notes: The letters “a”–“c” represent significant differences.

**Table 4:** Reductions of cumulative erosion modulus under different rainfall intensities (Jag S and Jag C162).

Reductions of Cumulative Erosion Modulus (%)							
Rainfall Intensity (mm/min)	Control (kg/m^2^)	Jag S Rates			Jag C162 Rates		
		1 g/m^2^	3 g/m^2^	5 g/m^2^	1 g/m^2^	3 g/m^2^	5 g/m^2^
1.0	0.14 c	19.1 c	35.1 bc	48.4 b	27.5 bc	56.7 ab	63.3 ab
1.5	0.33 b	30.5 ab	56.9 a	66.9 a	30.5 ab	56.9 ab	66.9 a
2.0	0.50 c	20.5 b	54.7 a	64.3 a	20.5 b	55.7 a	69.2 a

Notes: The letters “a”–“c” represent significant differences.
